# Effects of the biocide cybutryne (irgarol) on freshwater invertebrates

**DOI:** 10.7717/peerj.21773

**Published:** 2026-10-05

**Authors:** Jörg Oehlmann, Stefanie Schabio, Simone Ziebart, Stefan Meinecke, Matthias Oetken, Ulrike Schulte-Oehlmann

**Affiliations:** 1 Kompetenzzentrum Wasser Hessen, Frankfurt am Main, Germany; 2 Department Aquatic Ecotoxicology, Johann Wolfgang Goethe Universität Frankfurt am Main, Frankfurt am Main, Germany; 3 Department IV 2.5, German Environment Agency, Berlin, Germany

**Keywords:** Toxicity, Antifouling, Annelids, Arthropods, Mollusks, *Lumbriculus*, *Chironomus*, *Potamopyrgus*, Reproduction, Chronic tests

## Abstract

Cybutryne (irgarol) is a triazine biocide formerly used in antifouling paints worldwide and still detected in freshwater ecosystems in Europe, Asia, and North America despite its ban in antifouling paints in Europe in January 2017 and in the USA in April 2021. While primary producers are considered the most sensitive targets due to cybutryne’s mode of action as a photosystem II inhibitor, data on freshwater invertebrates—particularly from standardized chronic tests—remain scarce. In this study, we investigated acute and chronic effects of cybutryne on three freshwater invertebrates representing major taxonomic phyla: the midge *Chironomus riparius* (Arthropoda), the blackworm *Lumbriculus variegatus* (Annelida), and the freshwater mudsnail *Potamopyrgus antipodarum* (Mollusca). Acute toxicity tests showed low sensitivity, with immobilization observed only in *C. riparius* larvae at mg/L concentrations (48 h 50% effect concentration (EC_50_) = 2.96 mg/L), while no acute effects occurred in *L. variegatus* or *P. antipodarum* up to five mg/L. In chronic sediment-water tests, *C. riparius* exhibited no significant effects on survival, emergence, development time, sex ratio, or reproduction at concentrations up to five µg/L. In *L. variegatus*, reproduction was the most sensitive endpoint, with a 28-day 10% effect concentration (EC_10_) of 0.268 µg/L (95% confidence interval: 0–462 µg/L) and a lowest observed effect concentration (LOEC) of 0.8 µg/L. In contrast, chronic reproduction tests with *P. antipodarum* revealed pronounced and consistent increases in embryo numbers at all tested cybutryne concentrations (0.05–5 µg/L), resulting in inverted U-shaped concentration-response relationships that precluded derivation of a no observed effect concentration (NOEC) or EC_10_ values using conventional monotonic concentration-response models. Exploratory evaluation of quadratic hormesis-type models confirmed the non-monotonic inverted U-shaped concentration-response relationship. However, robust benchmark-concentration estimates could not be derived. The reproductive responses resembled those induced by estrogenic positive controls (17*α*-ethinylestradiol and bisphenol A) but the present results do not provide direct mechanistic evidence for endocrine disruption. Our results demonstrate that acute cybutryne exposure did not result in negative effects for the test organisms in the applied concentration range of up to five mg/L, except for *C. riparius*, while chronic reproductive endpoints—particularly in mollusks—appeared to be highly sensitive and suggest the possibility of endocrine-related or other non-specific reproductive modulation mechanisms that require further mechanistic investigation. These findings highlight the importance of including sensitive invertebrate reproduction tests in environmental risk assessment of antifouling biocides.

## Introduction

The triazine herbicide cybutryne (Irgarol 1051^®^, N-tert-butyl-N′-cyclopropyl-6-methylthio-1,3,5-triazine-2,4-diamine, CAS no. 28159-98-0) has been used as an effective biocide in antifouling coatings on ship hulls, harbor, and offshore installations and as an algicide in paints and plasters on external facades of buildings (Burkhardt et al., 2007). After the ban of tributyltin (TBT) by the International Maritime Organization (IMO) in January 2008, the use of antifouling paints containing copper and organic co-biocides (=booster biocides) such as cybutryne increased considerably (Mohr et al., 2009; Martins et al., 2018). These co-biocides usually made up 0.1 to 10% of the paint formulation and were expected not to occur in ambient water at concentrations to trigger acute toxic effects on non-target species (Martins et al., 2018).

Despite this expectation, adverse effects of cybutryne have been described in marine and freshwater ecosystems so that in Europe, Asia, and North America its use has been restricted or banned (Cresswell et al., 2006; Dafforn, Lewis & Johnston, 2011). In the European Union the use of cybutryne in antifouling paints was banned in January 2017 by Commission Decision (EU) 2016/107 (EC, 2016) and in the USA in April 2021 (Cash, 2021). In February 2020, the IMO agreed on a ban of cybutryne in antifouling systems from 1 July 2022 (DNV-GL, 2020).

Cybutryne is a potent photosystem (PS) II inhibitor, blocking photosynthetic electron transport by displacing plastoquinone from a specific-binding site on the D1 protein subunit of PS II (Jansen et al., 1993). This mode of action causes oxidative stress, including photo-oxidation of chlorophyll and cell necrosis so that primary producers are considered the most sensitive taxonomic group for cybutryne with typical EC_50_ values in the upper ng/L and lower µg/L range for target species (Mohr et al., 2009; Thomas & Brooks, 2010; Martins et al., 2018). In addition to effects on primary producers, cybutryne has also been associated with developmental and physiological alterations in vertebrates, including effects on cardiac and neurological functions (Konstantinou & Albanis, 2004).

Cybutryne is a white powder with a garlic-like odor, characterized by a low vapor pressure (3.4*10^−5^ Pa at 25 °C) and a low solubility in tap water (9.0 mg/L at pH 7 and 20 °C) which is further decreasing at higher salinity of water. The log *P*_OW_ of 3.1–3.2 indicates a potential for bioaccumulation with measured bioconcentrations factors ranging from 250 L/kg in fish to 10,560 L/kg in macrophytes (Mohr et al., 2009; ECHA, 2014). Cybutryne is recalcitrant to biodegradation and abiotic degradation in water is very slow, resulting in a half-life of 100 to 200 days (Martins et al., 2018). Sorption to suspended particles and sediments may contribute to the environmental persistence of cybutryne. The main metabolite M1 (also referred to as GS26575) is comparably persistent but more than 10 times less toxic to autotrophic organisms than cybutryne (Mohr et al., 2009; ECHA, 2014).

Due to its poor biodegradability, possible lingering inputs from historical applications and international shipping, cybutryne has been detected in water samples worldwide at concentrations up to 4.2 µg/L in coastal ports and marinas (Basheer, Tan & Lee, 2002), up to 7.6 µg/L in estuaries (Korkmaz et al., 2023) and in freshwater ecosystems at maximum concentrations of 2.4 µg/L (Lambert, Thomas & Davy, 2006). These measured concentrations (MECs) are considerably higher than calculated predicted environmental concentrations (PEC) of 0.421 µg/L inside harbors and 0.012 µg/L in the wider environment outside harbors in a realistic worst-case scenario according to ECHA (2014). This discrepancy between MECs and model-based exposure estimates suggests that site-specific emission sources, environmental conditions, or assumptions used in the PEC calculations do not fully capture real-world exposure scenarios.

Cybutryne is considered as a priority substance in annex I of the European Union Water Framework Directive with an environmental quality standard (EQS) of 2.5 ng/L as annual average (AA-EQS) and 16 ng/L as maximum allowable concentration (MAC-EQS), providing protection for freshwater ecosystems with regard to chronic and acute effects, respectively (EC, 2013). Considering more recent ecotoxicity data for cybutryne, Martins et al. (2018) derived an EQS of 0.22 ng/L for cybutryne in freshwater ecosystems, based on a probabilistic approach using species sensitivity distributions (SSDs) and applying an assessment factor (AF) of 5. The most sensitive primary producer in their data set was the common stonewort *Chara vulgaris* with a no observed effect concentration (NOEC) of two ng/L in a 14 d chronic test (Lambert, Thomas & Davy, 2006), while the most sensitive freshwater animal species was the rainbow trout *Oncorhynchus mykiss* with a NOEC of four µg/L in a 60 d chronic test with larvae (Mochida & Fujii, 2009). According to their assessment, no results for cybutryne from standard toxicity tests with invertebrates were available, except a 48 h EC_50_ of 5.3 mg/L and a lowest observable effect concentration (LOEC) of 2.4 mg/L with *Daphnia magna* (Mezcua et al., 2002; Mochida & Fujii, 2009) and a 24 h EC_50_ of 5.7 mg/L with *Daphnia pulex* (Okamura et al., 2000). The result of an acute non-standardized toxicity test with the crustacean *Thamnocephalus platyurus* indicated a slightly lower sensitivity with an LC_50_ of 12 mg/L (Okamura et al., 2000). More recently, Kim et al. (2024) identified that sub-lethal concentrations of cybutryne induced adverse effects across multiple generations in the water flea *Moina macrocopa*, leading to decreased survival and body length with a LOEC of 13 µg/L and to decreased reproductive output at 1.3 µg/L.

Mohr et al. (2008) and Mohr et al. (2009) reported a comparable sensitivity of primary producers and invertebrates in a 150 day study in indoor ponds. The EC_50_ for periphyton chlorophytes and macrophytes was 0.34 µg/L and 0.21 µg/L, respectively, while it was 0.09 µg/L and 0.11 µg/L for cyclopoid copepods and ostracods, respectively. In this mesocosm study, the pulmonate snail *Radix balthica* was analyzed (Umweltbundesamt, 2007). The main effects were reduced offspring per parent (EC_10_: 0.036 µg/L), reduced spermatogenesis (EC_10_: 0.040 µg/L), hypertrophy of the albumen gland (EC_10_: 0.058 µg/L) and reduced size of male genital organs (EC_10_: 0.069 µg/L). The study concluded that cybutryne is a reproductive toxicant for *R. balthica* at environmentally relevant concentrations. The specificity of effects on the male reproductive system and on spermatogenesis in the ng/L range were suggestive for a potential endocrine modulation by cybutryne. In this study, the reported outcome could be influenced by shortage of food, reduced water quality, possible presence of algal toxins as direct effects of the herbicide and by infestation with parasites.

On the other hand, there are indications of an increased sensitivity of mollusks to cybutryne including reproduction-related endpoints *in vivo*. Morley et al. (2004) reported a significantly reduced survival of the bladder snail *Physa fontinalis* infested by the trematode *Echinoparyphium recurvatum* under exposure to 10 µg cybutryne/L while mortality in non-infested snails was not increased. DeLorenzo et al. (2009) found a significantly reduced growth of the hard clam *Mercenaria mercenaria* when exposed to 1 and 10 µg cybutryne/L and Mai et al. (2013) reported a significant decline in fertilization success in the Pacific oyster *Crassostrea gigas* at one µg cybutryne/L and an increase in abnormal D-larvae prevalence when freshly fertilized embryos were treated with 0.01 µg cybutryne/L. A number of triazine herbicides, namely atrazine, are known endocrine disruptors (for review: Mnif et al., 2011). Moreira et al. (2018) have shown for cybutryne specifically that levels of the thyroid hormones T3 and T4 were altered in the inland silverside *Menidia beryllina* at concentrations of 0.1, 1 and 10 µg/L.

Given the still recent environmental relevance of cybutryne (Cui, Yang & Liu, 2022; Machate et al., 2021; Luo et al., 2023),the low number of available results from acute and chronic toxicity studies with invertebrates is noteworthy. It was therefore the aim of our study to determine effect concentrations for cybutryne in acute and chronic tests with the non-biting midge *Chironomus riparius*, the blackworm *Lumbriculus variegatus* and the freshwater mudsnail *Potamopyrgus antipodarum* as representatives of the three most species-rich invertebrate phyla (Arthropoda, Annelida, Mollusca) in freshwater ecosystems under standardized conditions in the laboratory. We hypothesized that (1) the invertebrates show a sensitivity comparable to that of primary producers in chronic tests and (2) their reproduction in particular is negatively affected by cybutryne.

## Materials & Methods

### Chemicals

Cybutryne (CAS no. 28159-98-0, Pestanal^®^ analytical standard) was purchased from Sigma-Aldrich (Schnelldorf, Germany). In the chronic reproduction test with *Potamopyrgus antipodarum*, the estrogenic compounds 17*α*-ethinylestradiol (EE2, CAS no. 57-63-6, >98% purity, Sigma-Aldrich) and bisphenol A (BPA, CAS no. 80-05-7, >97% purity, Merck, Schuchardt, Germany) as well as the xeno-androgen TBT chloride (CAS no. 1461-22-9, 96% purity, Sigma-Aldrich) were used as positive controls. Ethanol (CAS no. 64-17-5, analytical grade, Sigma-Aldrich) and dimethyl sulfoxide (DMSO, CAS no. 67-68-5, ≥99.9% purity, Sigma-Aldrich) served as solvents at a final concentration of 0.01% (v/v).

### Chemical analysis

Cybutryne and its metabolite M1 were quantified in the exposure media according to Mohr et al. (2008). Briefly, unfiltered one L water samples were collected, spiked with propazine (CAS no. 139-40-2, Pestanal^®^ analytical standard, Sigma-Aldrich) as an internal standard, extracted using Isolute ENV+solid-phase extraction columns (Biotage, Uppsala, Sweden), and analyzed by GC-MS in selected ion monitoring mode using Hewlett-Packard (Palo Alto, CA, USA) and Agilent GC-MS systems (Santa Clara, CA, USA). Mean recovery rates were 101% and 102% for cybutryne and M1, respectively. Limits of detection (LOD) were 5 and 4 ng/L and limits of quantification (LOQ) were 16 and 11 ng/L for cybutryne and M1, respectively.

In the chronic tests with *Chironomus riparius* and *Lumbriculus variegatus*, cybutryne concentrations were measured in pooled exposure media at test termination. Reported analytical replicates refer to independent chemical determinations of pooled samples generated from multiple biological replicates. In the *P. antipodarum* reproduction tests, concentrations were determined throughout the 56-day exposure period and time-weighted mean concentrations were calculated according to Organisation for Economic Co-operation and Development (OECD) guideline 211 (OECD, 2012). Sediments and feed were not analyzed.

### Acute toxicity tests

Acute toxicity tests were conducted with first-instar larvae of *C. riparius* and adult *L. variegatus* and *P. antipodarum* from laboratory cultures following OECD guideline 235 (OECD, 2011), with two modifications: (1) 24 individuals were tested individually instead of 20 animals in four replicates, and (2) 24-well microtiter plates containing two mL reconstituted water (OECD, 2016) per well were used instead of larger test vessels. Test organisms were exposed to nominal cybutryne concentrations of 10–5,000 µg/L plus a negative control. Exposure lasted 48 h for *C. riparius* and 7 d for *L. variegatus* and *P. antipodarum*. Water was the only solvent. Exposure media were not changed, and animals were not fed during exposure. Immobilization was assessed as a proxy for mortality according to OECD criteria.

### Chronic toxicity tests

Stock solutions were freshly prepared for each experiment and stored protected from light until use. All test organisms originated from in-house laboratory cultures maintained at Goethe University Frankfurt. Physico-chemical water quality parameters were monitored throughout the experiments, and test vessels were randomly distributed within the climate-controlled rooms.

### Chironomus riparius

The sediment-water toxicity test was conducted according to OECD guideline 219 (OECD, 2023). Four replicates containing 20 first-instar larvae each were exposed to nominal cybutryne concentrations of 0.13, 0.32, 0.8, 2, and 5 µg/L. Artificial sediment and reconstituted water were prepared according to OECD specifications. The test was performed at 20 °C with a 16:8 h light:dark cycle. Water was the only solvent. Exposure media were not changed over the course of the experiment, but deionised water was added twice a week to compensate for evaporation losses. Animals were fed every second day with finely ground TetraMin^®^ (Tetra GmbH, Melle, Germany). Emergence success and mean emergence time (EmT_50_) were determined as primary endpoints (OECD, 2023). Sex ratio and clutches per female were additionally recorded following OECD guideline 233 (OECD, 2010).

### Lumbriculus variegatus

The sediment-water toxicity test was performed according to OECD guideline 225 (OECD, 2007) with the test substance being spiked into the water phase. Four replicates containing 10 synchronized worms of identical development stage were exposed to the same cybutryne concentrations as used for *C. riparius* in 200 mL OECD 242 medium (OECD, 2016) plus 50 g artificial sediment (OECD, 2007) in 250 mL glass beakers. The test was conducted at 20 °C under a 16:8 h light:dark cycle. Water was the only solvent. The exposure media were not changed over the course of the experiment, but deionised water was added twice a week to compensate for evaporation losses. The animals were fed every second day with finely ground TetraMin^®^ (Tetra GmbH, Melle, Germany). At test termination, worms were counted and mean individual dry weight was determined.

### Potamopyrgus antipodarum

Reproduction tests were conducted according to OECD guideline 242 (OECD, 2016). A preliminary experiment (test series I) compared semi-static and flow-through exposure scenarios over 56 days. Because measured concentrations in the semi-static system agreed more closely with nominal concentrations, this exposure regime was selected for the definitive experiment (test series II). Details of test series I are provided in the Supplemental Information.

In test series II, four replicates containing 25 adult snails each (≥3.5 mm shell height) were exposed for 56 days to nominal cybutryne concentrations of 0.05, 0.13, 0.32, 0.8, 2, and 5 µg/L. In addition to the negative control (NC) and solvent control (SC; DMSO concentration: 0.01% [v/v]), positive controls included the estrogenic compounds BPA (five µg/L) and EE2 (25 ng/L) as well as the androgenic and reprotoxic compound TBT (250 ng as Sn/L). DMSO was used as a solvent for the application of cybutryne and the positive control substances. The test was conducted at 16 °C under a 16:8 h light:dark cycle with semi-static renewal twice weekly. The animals were fed three times a week with finely ground TetraMin^®^ (Tetra GmbH, Melle, Germany). On days 28 and 56, shell dimensions and embryo numbers in the brood pouch were determined in 10 randomly selected snails per replicate.

### Statistical analyses

Data analysis and visualization were performed using GraphPad Prism^®^ (Version 5.03, GraphPad Software LLC, Boston, MA, USA) and ToxRat^®^ Professional (Version 3.3.0, ToxRat-Solutions GmbH, Alsdorf, Germany). Quantal endpoints were analyzed using Fisher’s exact test. Continuous endpoints were analyzed using one-way analysis of variance (ANOVA) followed by Dunnett’s multiple comparison test if assumptions of normality and homogeneity of variance were met; otherwise, Kruskal-Wallis tests followed by Dunn’s multiple comparison test were applied. Negative and solvent controls were pooled when no statistically significant differences were detected (Green & Wheeler, 2013).

Concentration–response curves with 10% and 50% effect concentrations (EC_10_, EC_50_) were derived using four-parameter logistic regression models. Because embryo production in *P. antipodarum* exhibited non-monotonic concentration–response relationships, conventional EC_X_ modeling was not appropriate. Exploratory evaluation using alternative non-monotonic models indicated substantial model uncertainty and produced multiple benchmark-concentration solutions. Therefore, concentration–response relationships for this endpoint were interpreted descriptively and discussed in context of limitations of threshold-based approaches. Statistical significance was defined as *p* < 0.05 and significance levels are indicated by asterisks: *p < 0.05, **p < 0.01, and ***p < 0.001.

## Results

### Acute toxicity tests

All acute toxicity tests fulfilled the validity criteria of OECD guideline 235 (OECD, 2011). In the test with *Chironomus riparius*, cybutryne induced a concentration-dependent increase in larval mortality. The 24 h EC_50_ was 4,790 µg/L (95% confidence interval [CI]: 2,580–8,890 µg/L), decreasing to 2,960 µg/L (95% CI [1,310–6,670] µg/L) after 48 h (Fig. 1).

**Figure 1:**
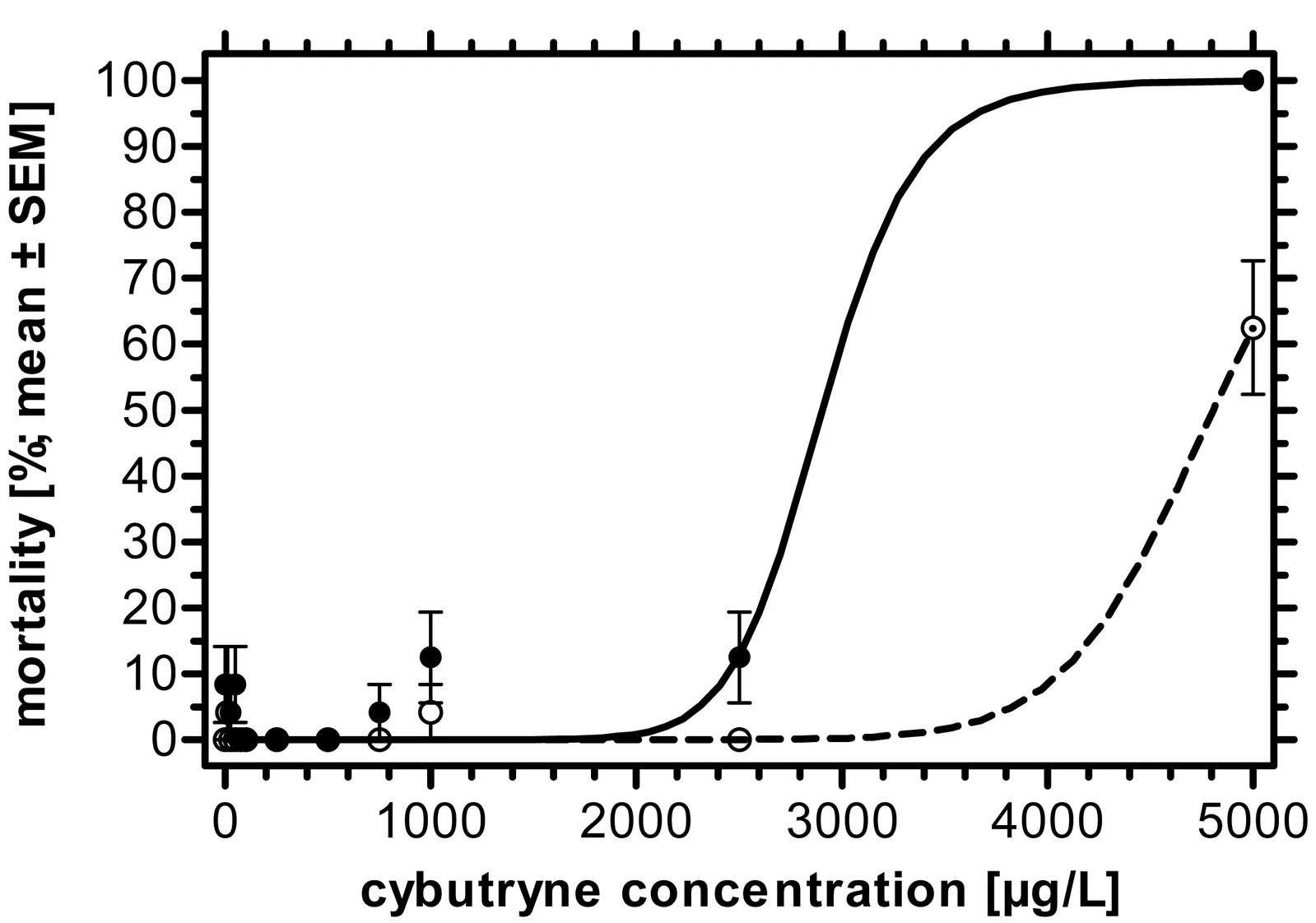
Acute toxicity of cybutryne in the test with *Chironomus riparius* according to OECD guideline 235 (OECD, 2011). Data are presented as mean mortality in % (±standard error of the mean, SEM) and calculated concentrations response curves after 24 (open circles, dashed line) and 48 h (filled circles, solid line).

In contrast, no effects were observed in *Lumbriculus variegatus* or *Potamopyrgus antipodarum* within the tested concentration range of 10–5,000 µg/L. All animals survived the 7-day exposure period.

### Chronic toxicity tests

### Chironomus riparius

Measured cybutryne concentrations at test termination ranged from 94% to 100% of nominal concentrations (Table 1). The main metabolite M1 was detected only in the 2 and 5 µg/L treatment groups at concentrations not exceeding 0.03 and 0.06 µg/L, respectively (Supplemental Information).

**Table 1:** Measured aqueous cybutryne concentrations in the chronic test with *Chironomus riparius* on termination of the experiment (µg/L; *n* = 2 independent determinations of pooled exposure media). Limit of detection: 0.005 µg/L; limit of quantification: 0.016 µg/L. n.d., not detected (below detection limit); NC, negative control.

Nominal concentrations	NC	0.13	0.32	0.8	2	5
Measured concentrations						
Range (min.–max.)	n.d.	0.12–0.14	0.27–0.33	0.79–0.80	1.93–2.07	4.53–5.28
Mean ± SD	n.d.	0.13 ± 0.01	0.30 ± 0.02	0.80 ± 0.01	2.00 ± 0.06	4.90 ± 0.31
% of nominal	–	100	94	100	100	98

The validity criteria (OECD, 2023) were met: Emergence in the negative control was 78.7%, emergence occurred between days 12 and 23, dissolved oxygen concentrations exceeded 60% air saturation, pH values remained within the acceptable range (6–9), and temperature variation did not exceed 1.0 °C.

No statistically significant effects of cybutryne were observed for any of the measured endpoints at concentration between 0.13 and 5 µg/L. Mortality ranged from 20.0 and 25.0% compared with 21.3% in the negative control. Mean emergence times (EmT_50_) varied between 19.1 and 21.6 d for males and between 20.7 and 23.7 d for females, compared with 19.3 d and 22.3 d in the negative control, respectively. Reproductive output ranged from 0.70 to 0.81 clutches per female compared with 0.68 in the control group. The proportion of females ranged from 50.0–67.1% and did not differ significantly from the expected 1:1 sex ratio.

### Lumbriculus variegatus

Measured cybutryne concentrations ranged from 93% to 100% of nominal values at test termination (Table 2). The metabolite M1 was detected only in the 2 and 5 µg/L treatment groups at concentrations ≤0.04 and ≤0.09 µg/L, respectively (Supplemental Information).

**Table 2:** Measured aqueous cybutryne concentrations in the chronic test with *Lumbriculus variegatus* on termination of the experiment (µg/L; *n* = 2 independent determinations of pooled exposure media). Limit of detection: 0.005 µg/L; limit of quantification: 0.016 µg/L. n.d., not detected (below detection limit); NC, negative control.

Nominal concentrations	NC	0.13	0.32	0.8	2	5
Measured concentrations						
Range (min.–max.)	n.d.	0.12–0.14	0.31–0.32	0.71–0.78	1.64–2.27	4.68–4.97
Mean ± SD	n.d.	0.13 ± 0.01	0.32 ± 0.05	0.74 ± 0.03	1.95 ± 0.26	4.86 ± 0.13
% of nominal	–	100	100	93	98	97

The validity criteria (OECD, 2007) were met.

Cybutryne exposure resulted in reduced worm numbers at all concentrations relative to the control, although statistically significant reductions occurred only at 0.8 and 5 µg/L (Fig. 2A). The calculated EC_10_ for reproduction was 0.268 µg/L cybutryne (95% CI [0–462] µg/L), while the NOEC and LOEC were 0.32 µg/L and 0.8 µg/L, respectively. In contrast, mean individual dry weight increased in all exposure groups and reached statistical significance only at five µg/L (Fig. 2B), resulting in a NOEC and LOEC of 2 and 5 µg/L, respectively. An EC_10_ could not be derived for this endpoint.

**Figure 2:**
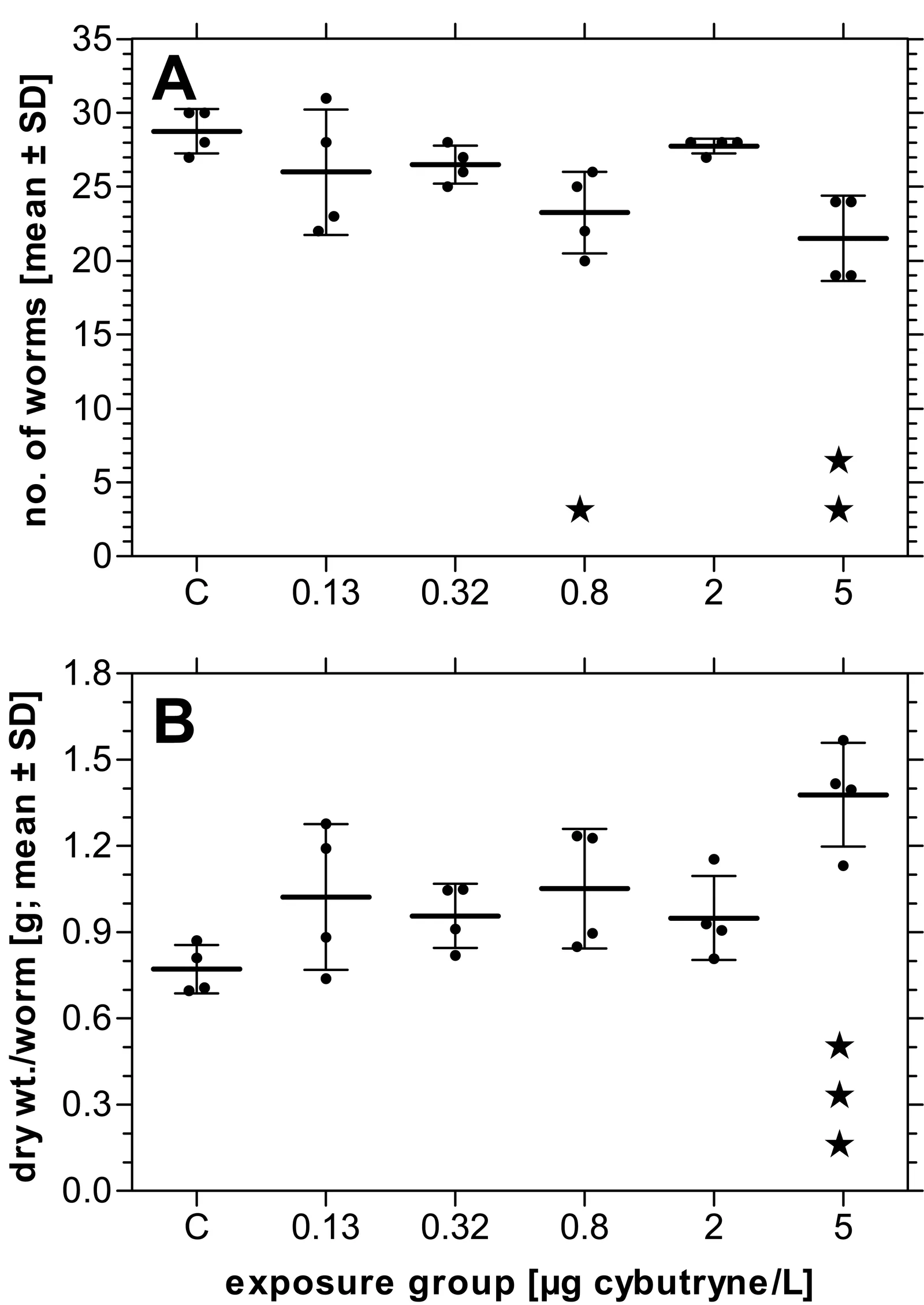
Effects of cybutryne in the chronic toxicity test with *Lumbriculus variegatus* according to OECD guideline 225 (OECD, 2007) with the test substance being spiked into the water phase. (A) Number of worms and (B) dry weight of worms at the end of the test on day 28. Data are presented as values per replicate (dots) and mean values per treatment (bold horizontal line) ± standard deviation (SD, error bars). Asterisks indicate significant differences from the control (C) using one-way ANOVA followed by Dunnett’s multiple comparison test: *p < 0.05, **p < 0.01, and ***p < 0.001.

### Potamopyrgus antipodarum

In the preliminary experiment (test series I), embryo production in control groups did not differ significantly between the semi-static and flow-through exposure scenarios. Cybutryne-induced reproductive responses were observed both in the flow-through system and in the semi-static design. Because analytical verification demonstrated better agreement between measured and nominal concentrations in the semi-static exposure scenario, this design was selected for the definitive experiment. Detailed results of test series I are provided in the Supplemental Information.

In the definitive experiment (test series II), time-weighted mean cybutryne concentrations ranged from 90% to 106% of the nominal concentrations over the 56-day test period (Table 3). The metabolite M1 was detected only in the five µg/L treatment in weeks 6 and 8 of the experiment at concentrations < 0.02 µg/L (Supplemental Information).

**Table 3:** Comparison of nominal and measured aqueous cybutryne concentrations in the chronic test series II with *Potamopyrgus antipodarum* over the course of the 56 day experiment (in µg/L, *n* = 8). Limit of detection: 0.005 µg/L; limit of quantification: 0.016 µg/L. n.d., not detected (below detection limit); NC, negative control.

Nominal concentrations	NC	0.05	0.13	0.32	0.8	2	5
Measured concentrations							
Range (min.–max.)	n.d.	0.04–0.05	0.10–0.14	0.28–0.32	0.71–0.82	1.80–2.17	4.92–6.25
Mean ± SD	n.d.	0.05 ± 0.003	0.12 ± 0.02	0.30 ± 0.01	0.78 ± 0.03	1.97 ± 0.11	5.33 ± 0.46
Time-weighted mean^a^	–	0.048	0.117	0.304	0.778	1.97	5.32
% of nominal^b^	–	96	90	95	97	98	106

**Notes.**

a Calculated according to OECD guideline 211, annex 6 (OECD, 2012).

b Referring to measured time-weighted mean concentration.

The validity criteria (OECD, 2016) were fulfilled. Mortality in the control group was 0%, mean number of embryos exceeded the required minimum of five embryos per female at the end of the test, dissolved oxygen concentrations remained above 60% air saturation, and mean water temperature was maintained 16 ± 1.5 °C throughout the experiment.

The positive controls confirmed the responsiveness of the test system (Fig. 3). Embryo numbers increased in the estrogenic positive controls containing bisphenol A (five µg/L) by 68.0% and 106% compared to the control after 28 and 56 days, respectively, and in the 17*α*-ethinylestradiol treatment (25 ng/L) by 157% and 178%, respectively. In contrast, tributyltin (250 ng as Sn/L), used as a xeno-androgen and reproductive toxicant control, reduced embryo numbers by 54.1% and 84.1% after 28 and 56 days, respectively.

**Figure 3:**
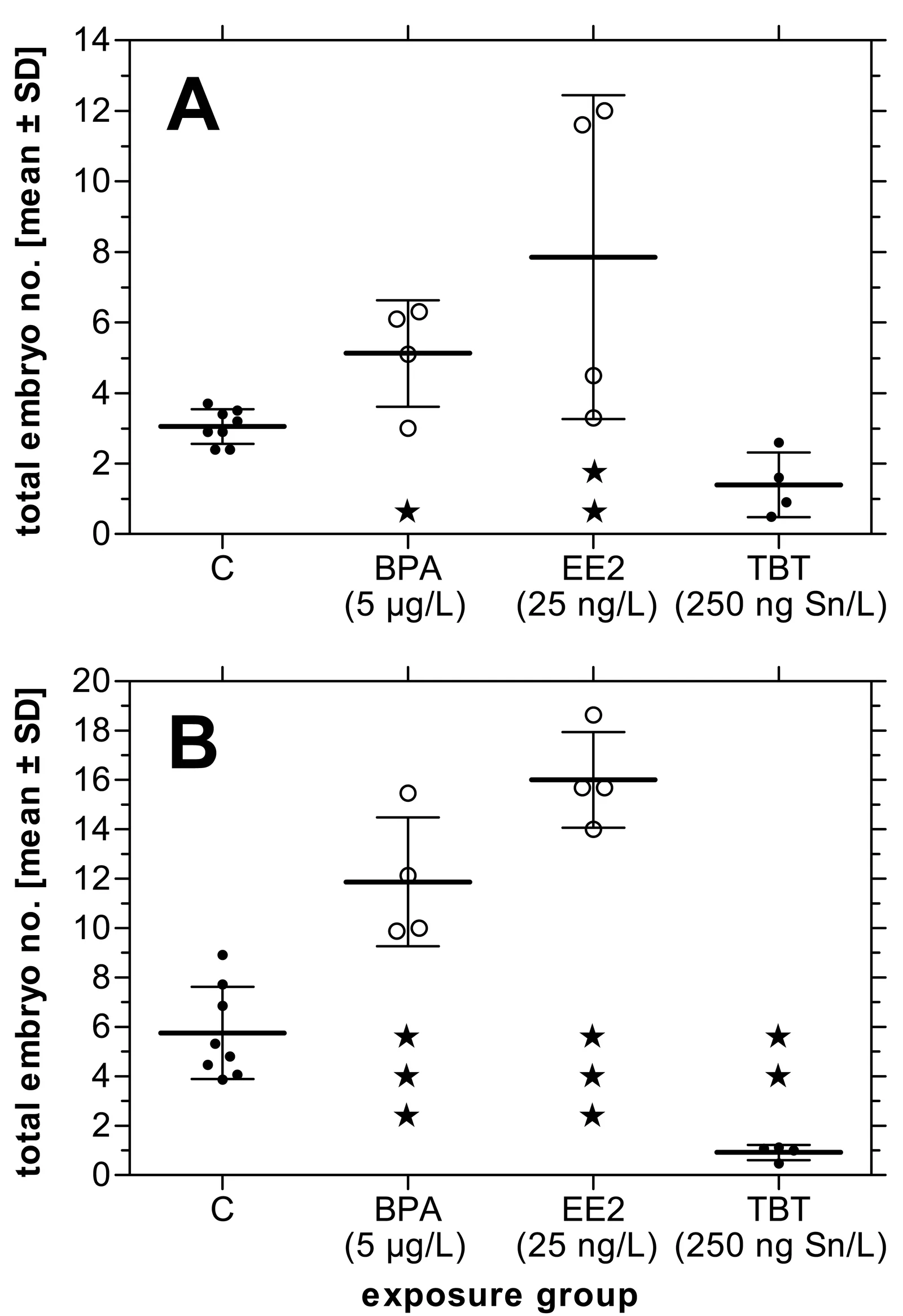
Effects of positive controls on the number of embryos in the brood pouch of *Potamopyrgus antipodarum* in the chronic test series II. (A) Results after 28 days and (B) after 56 days. Data are presented as mean values per replicate (dots) and mean values per treatment (bold horizontal line) ± standard deviation (SD, error bars). C is the pooled negative and solvent control group, BPA, bisphenol A; EE2, 17*α*-ethinylestradiol; TBT, tributyltin. Asterisks indicate significant differences from the control (C) using one-way ANOVA followed by Dunnett’s multiple comparison test: *p < 0.05, **p < 0.01, and ***p < 0.001.

Cybutryne exposure induced significant increases in embryo numbers already at the lowest tested concentration of 0.05 µg/L both after 28 and after 56 days (Fig. 4). Consequently, a NOEC could not be derived. The concentration–response relationship was non-monotonic and exhibited an inverted U-shaped pattern at both sampling times. Maximum responses occurred at 0.32 µg/L after 28 days, with embryo numbers increasing by 200% relative to the control, and at 0.13 µg/L after 56 days, with an increase of 112%.

**Figure 4:**
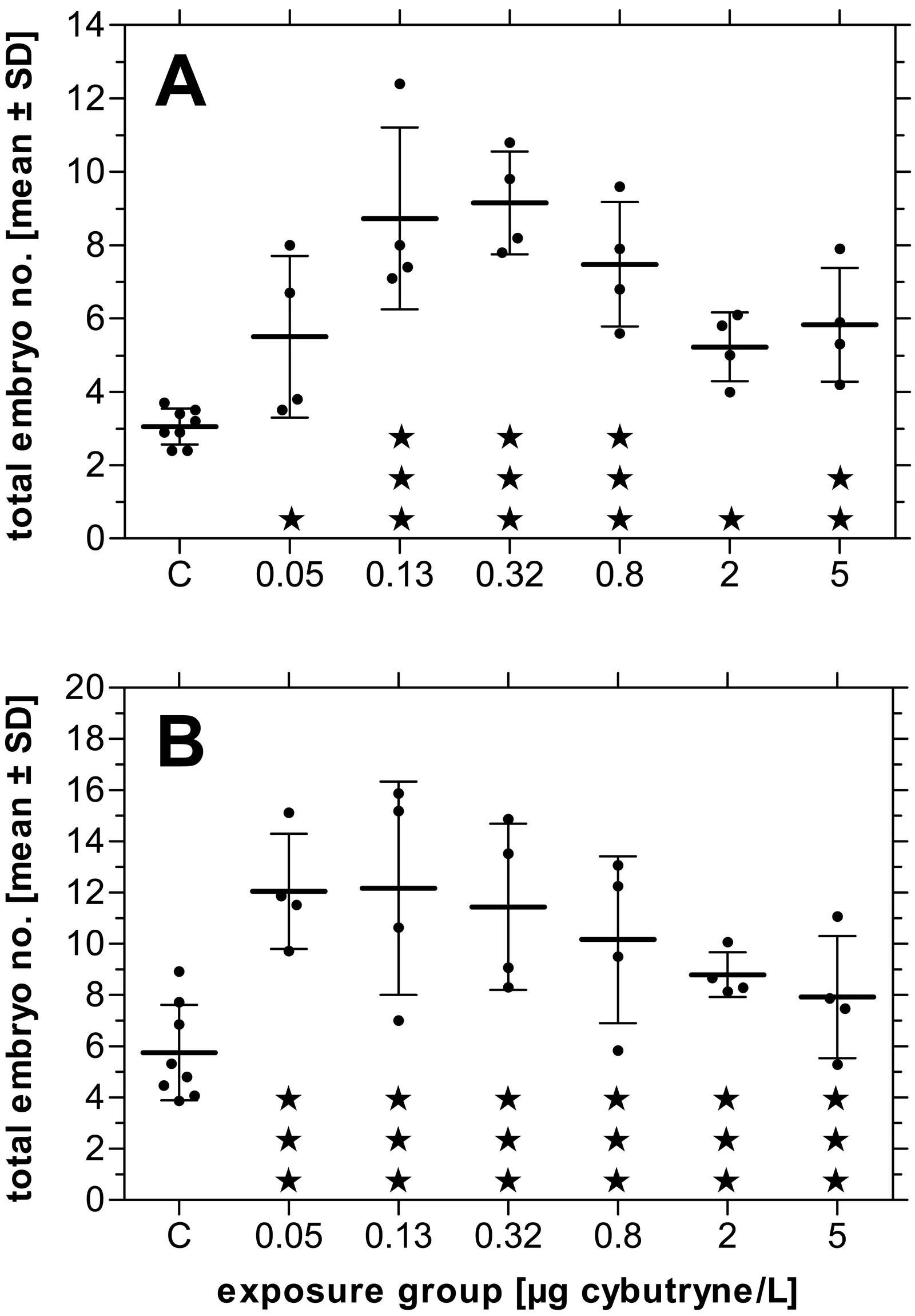
Effects of cybutryne on the number of embryos in the brood pouch of *Potamopyrgus antipodarum* in the chronic test series II. (A) Results after 28 days and (B) after 56 days. Data are presented as mean values per replicate (dots) and mean values per treatment (bold horizontal line) ± standard deviation (SD, error bars). C is the pooled negative and solvent control group. Asterisks indicate significant differences from the control (C) using one-way ANOVA followed by Dunnett’s multiple comparison test: * *p* < 0.05, ** *p* < 0.01, and *** *p* < 0.001.

## Discussion

The present study provides a comprehensive assessment of the acute and chronic effects of the antifouling biocide cybutryne on freshwater invertebrates representing three major taxonomic phyla. Contrary to our first hypothesis, the tested invertebrates did not generally exhibit a sensitivity comparable to that of primary producers in standard chronic toxicity tests. Our second hypothesis was partially supported because pronounced reproductive effects occurred in *Potamopyrgus antipodarum*, whereas reproductive endpoints in *Chironomus riparius* and *Lumbriculus variegatus* were less sensitive.

### Acute toxicity of cybutryne to freshwater invertebrates

Acute toxicity tests demonstrated a generally low sensitivity of the tested invertebrates to cybutryne. *C. riparius* larvae showed concentration-dependent immobilization, with 48 h EC_50_ values in the mg/L range, consistent with previous findings for cladocerans (*Daphnia magna*, *D. pulex*) and other crustaceans (Kim et al., 2024; Mezcua et al., 2002; Mochida & Fujii, 2009; Okamura et al., 2000). In contrast, no acute effects were observed in *L. variegatus* or *P. antipodarum* up to the highest tested concentration of five mg/L. These results confirm that cybutryne does not exert strong acute toxicity on freshwater invertebrates and that acute endpoints are unlikely to be protective for chronic exposure to this compound.

The low acute toxicity is consistent with cybutryne’s primary mode of action as a photosystem II inhibitor, which directly targets photosynthetic organisms rather than heterotrophic consumers (Jansen et al., 1993; Mohr et al., 2009). Accordingly, acute mortality in invertebrates likely reflects nonspecific toxicity occurring at high concentrations far exceeding those measured in the environment.

### Chronic effects in sediment-dwelling invertebrates

In chronic sediment-water tests, *C. riparius* showed no adverse effects on survival, emergence, development time, sex ratio, or reproduction up to five µg/L. This finding contrasts with the results of Mohr et al. (2008), who reported high sensitivity of other arthropods (cyclopoid copepods and ostracods) in a mesocosm experiment over 150 days at sub-µg/L concentrations. The discrepancy may reflect differences in species sensitivity, exposure routes and duration, or indirect food-web mediated effects present in mesocosms but absent under controlled laboratory conditions. Our results suggest that, under standardized test conditions, *C. riparius* is comparatively insensitive to cybutryne, with effect concentrations up to an order of magnitude higher than current freshwater EQS values.

In *L. variegatus*, reproduction was the most sensitive endpoint, with a calculated EC_10_ of 0.268 µg/L and a LOEC of 0.8 µg/L. The EC_10_ estimate should be interpreted cautiously because the associated confidence interval is broad and poorly constrained. These concentrations overlap with measured environmental concentrations reported for freshwater systems (Cui, Yang & Liu, 2022; Lambert, Thomas & Davy, 2006) and with predicted environmental concentrations for harbor environments (ECHA, 2014). The concomitant increase in individual body weight at higher concentrations may indicate a density-dependent response, where reduced population size leads to increased food availability per individual rather than a direct stimulatory effect of cybutryne. Similar trade-offs between reproduction and growth have been observed in oligochaetes exposed to other stressors and are commonly interpreted as energy reallocation rather than reproductive modulation (Paumen et al., 2008; Woermann, Kontchou & Sures, 2021).

Overall, the chronic effects observed in *L. variegatus* indicate a moderate sensitivity to cybutryne, higher than in *C. riparius* but still markedly lower than observed in primary producers.

### Reproductive effects in *Potamopyrgus antipodarum*

The most striking and ecologically relevant finding of this study is the pronounced reproductive response observed in *P. antipodarum*. In both test series, cybutryne exposure resulted in significant increases in embryo numbers at environmentally relevant concentrations, including the lowest tested concentration of 0.05 µg/L. The observed inverted U-shaped concentration–response relationships indicate a non-monotonic response pattern that cannot be adequately described using conventional monotonic concentration–response models such as the four-parameter logistic model applied in the present study. Under such conditions, threshold-based approaches including NOEC derivation have important limitations, and alternative approaches such as benchmark concentrations, spline-based, quadratic, or hormesis models may be more appropriate (Weltje, Vom Saal & Oehlmann, 2005). Exploratory quadratic hormesis-type modeling confirmed the non-monotonic inverted U-shaped response pattern. However, benchmark-concentration calculations based on a 10% deviation from controls produced multiple solutions because the response curve crossed the benchmark level on both the ascending and descending limbs. The estimated response maximum varied between sampling times and exposure scenarios, indicating that model-derived benchmark values were highly sensitive to model specification. More sophisticated hormesis models such as Brain-Cousens or Cedergreen models were considered, but the experimental design was not optimized for quantitative characterization of non-monotonic concentration–response relationships and model-derived threshold estimates were highly sensitive to model choice. We therefore refrained from reporting quantitative benchmark concentrations and interpret the response pattern descriptively.

The reproductive responses observed in *P. antipodarum* under cybutryne exposure resemble those induced by estrogenic compounds (Duft et al., 2007; Oehlmann et al., 2007; Sieratowicz et al., 2011). The positive controls with EE2 and BPA produced significant increases in embryo numbers, whereas TBT markedly reduced reproduction, confirming the responsiveness of the test system to endocrine-active and reproductive toxicants. The reproductive stimulation observed under cybutryne exposure resembles responses induced by estrogenic compounds but does not provide direct mechanistic evidence that cybutryne acts through endocrine receptor-mediated pathways. Increased embryo numbers may alternatively reflect altered reproductive timing, embryo retention, indirect physiological stress responses, or changes in energy allocation. TBT is known to act through multiple endocrine-related mechanisms, including interactions with cytochrome P450 enzymes and nuclear receptor pathways (Stange, Sieratowicz & Oehlmann, 2012). Although triazine compounds have been associated with endocrine activity in vertebrates (Mnif et al., 2011; Moreira et al., 2018), the present study did not include mechanistic endpoints and therefore cannot demonstrate endocrine disruption according to current regulatory criteria. Moreover, reproductive and gonadal effects of cybutryne in mollusks have previously been reported at environmentally relevant concentrations (Morley et al., 2004; Mai et al., 2013).

The lower baseline embryo numbers observed in test series II compared with test series I likely reflect natural biological variability between independently conducted experiments using different cohorts of animals at different seasons of the year (summer in series I, winter in series II) and slightly different experimental conditions, including solvent system, animal density, and vessel configuration (Geiß, Ruppert & Oehlmann, 2017).

The discrepancy between the semi-static and flow-through exposure scenarios observed in test series I further highlights the sensitivity of reproductive endpoints to exposure dynamics. Higher variability and more pronounced effects in the flow-through system may reflect larger deviations from nominal concentrations and thus differences in exposure stability, bioavailability, or stress related to hydrodynamic conditions. Nevertheless, the consistency of the stimulatory reproductive response across test series and exposure designs strongly suggests a substance-related effect.

### Environmental and regulatory implications

Despite regulatory activities to restrict the use of cybutryne in recent years, this biocide continues to be highly relevant to the environment. Machate et al. (2021) identified contamination of sediments with the antifouling biocides cybutryne and diuron as major drivers limiting the recovery of macrophyte communities in impaired lakes in Northern Germany. The analysis of six antifouling biocides in coastal waters by Luo et al. (2023) revealed the highest acute and chronic risk for cybutryne, well ahead of tributyltin and diuron. This finding was confirmed by Durak et al. (2021) for rivers in Poland and by Cui, Yang & Liu (2022) for coastal waters in China, Japan, Korea and Singapore and rivers in UK and USA.

From an environmental risk assessment perspective, the present results are highly relevant. The reproductive effects observed in *P. antipodarum* occurred at concentrations several orders of magnitude above current EQS values (ng/L range) but within the range of concentrations reported from contaminated freshwater systems (Lambert, Thomas & Davy, 2006; Machate et al., 2021). Importantly, the absence of a NOEC and the non-monotonic concentration–response relationship challenge traditional risk assessment approaches that rely on threshold-based endpoints.

Our findings support earlier indications from mesocosm studies that invertebrates, particularly mollusks, may be more sensitive to cybutryne than previously assumed based on standard laboratory tests (Mohr et al., 2008; Umweltbundesamt, 2007). The strong reproductive response in *P. antipodarum* suggests that this species is a suitable and sensitive test organism for detecting sublethal and potentially endocrine-mediated effects of cybutryne and similar biocides. The pronounced reproductive sensitivity observed in *P. antipodarum* may also indicate potential risks for other mollusks, including ecologically and economically important bivalve species.

## Conclusions

In conclusion, this study demonstrates that while acute toxicity of cybutryne to freshwater invertebrates is low, chronic exposure can induce substantial reproductive alterations in mollusks at environmentally relevant concentrations. The observed non-monotonic concentration–response relationships highlight limitations of conventional threshold-based risk assessment approaches for this type of response pattern. Although the reproductive stimulation observed in *P. antipodarum* was consistent with responses known for endocrine-active compounds, further mechanistic studies are required before endocrine disruption can be concluded. Incorporating sensitive invertebrate reproduction tests into environmental risk assessment frameworks may improve the protection of freshwater ecosystems from antifouling biocides and related compounds.

##  Supplemental Information

10.7717/peerj.21773/supp-1 Figure S1 Effects of cybutryne on the number of embryos in the brood pouch of *Potamopyrgus antipodarum* after 28 days in the chronic test series I (A) Test with semi-static and (B) with flow-through design. Data are presented as mean values per replicate (dots) and mean values per treatment (bold horizontal line) ± standard deviation (SD, error bars). C is the pooled negative and solvent control group, PC the positive control (30 ng 17*α*-ethinylestradiol/L). Asterisks indicate significant differences from the control (C) using one-way ANOVA followed by Dunnett’s multiple comparison test: *p < 0.05, **p < 0.01, and ***p < 0.001.

10.7717/peerj.21773/supp-2 Figure S2 Effects of cybutryne on the number of embryos in the brood pouch of *Potamopyrgus antipodarum* after 56 days in the chronic test series I (A) Test with semi-static and (B) with flow-through design. Data are presented as mean values per replicate (dots) and mean values per treatment (bold horizontal line) ± standard deviation (SD, error bars). C is the pooled negative and solvent control group, PC the positive control (30 ng 17*α*-ethinylestradiol/L). Asterisks indicate significant differences from the control (C) using one-way ANOVA followed by Dunnett’s multiple comparison test: *p < 0.05, **p < 0.01, and ***p < 0.001.

10.7717/peerj.21773/supp-3 Table S1 Comparison of nominal and measured aqueous cybutryne concentrations in the chronic test series I with *Potamopyrgus antipodarum* over the course of the 56 day experiment (in µg/L, *n* = 6) Limit of detection: 0.005 µg/L; limit of quantification: 0.016 µg/L. n.d. = not detected (below detection limit); NC = negative control.

10.7717/peerj.21773/supp-4 Supplemental Information 4 Results of all cybutryne analyzes in exposure media during the chronic tests and of the analyzed endpoints in the acute and chronic test series with the three invertebrate species

## Additional Information and Declarations

### Data Availability

The following information was supplied regarding data availability: The raw data are available in the Supplemental Files.
