# Supplementary material for: Effects of the biocide cybutryne (irgarol) on freshwater invertebrates
Source: PeerJ. 2026 Oct 5;14:e21773. doi: 10.7717/peerj.21773 (PMC13648218; doi:10.7717/peerj.21773)
Supplement: Supplementary file 2 [file peerj-14-21773-s002.pdf]

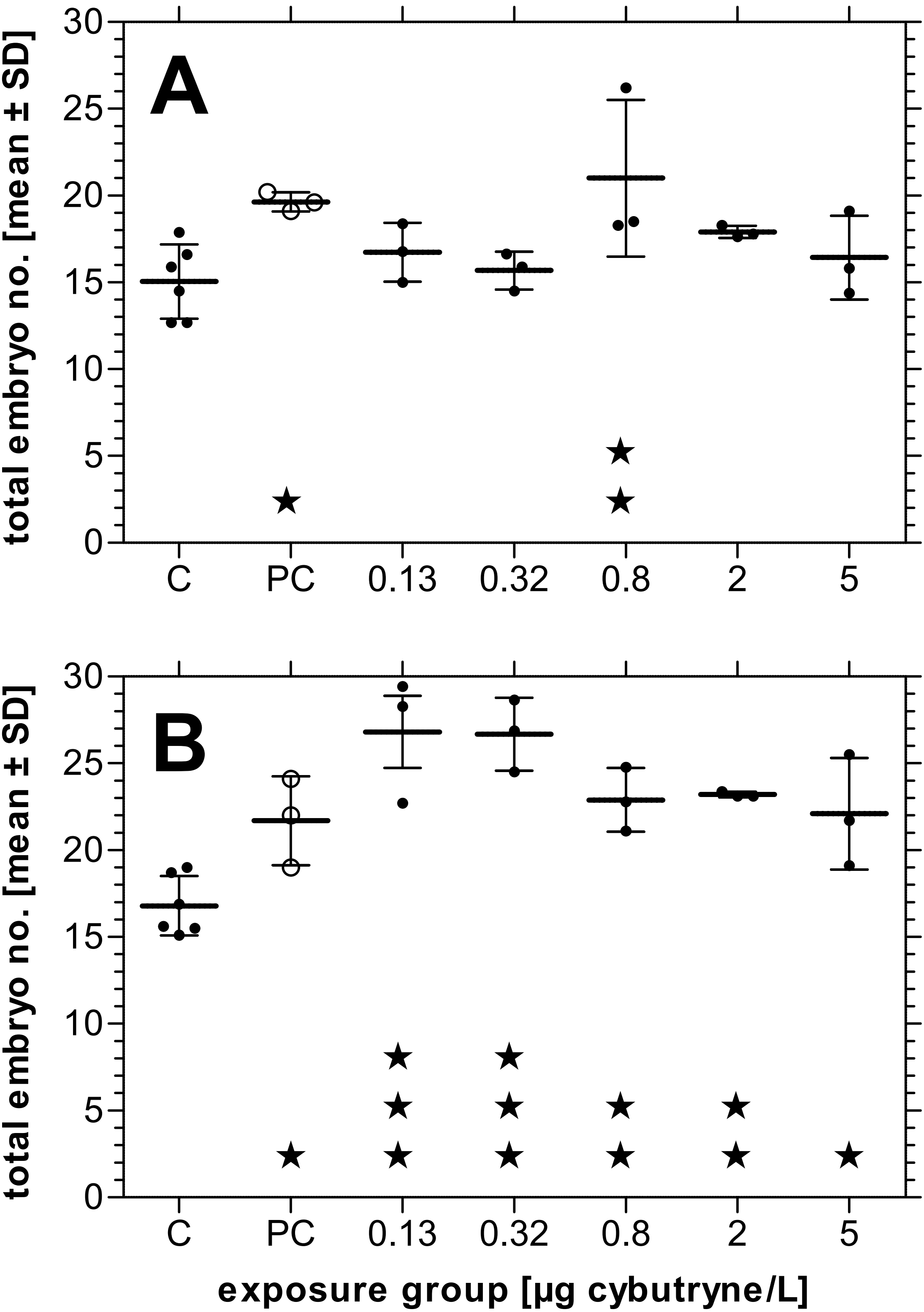

A
B
total embryo no. [mean ± SD]
30
25
20
15
10
5
0
C
PC
0.13
0.32
0.8
2
5
exposure group [µg cybutryne/L]
