# Supplementary material for: Effects of the biocide cybutryne (irgarol) on freshwater invertebrates
Source: PeerJ. 2026 Oct 5;14:e21773. doi: 10.7717/peerj.21773 (PMC13648218; doi:10.7717/peerj.21773)
Supplement: Supplementary file 3 [file peerj-14-21773-s003.docx]

Nominal concentrations	NC	0.13	0.32	0.8	2	5

Measured concentrations
**semi-static test**
range (min. - max.)	n.d.	0.14 – 0.16	0.25 – 0.37	0.64 – 0.83	1.57 – 1.99	3.56 – 4.93
mean ± SD	n.d.	0.15 ± 0.01	0.31 ± 0.04	0.75 ± 0.08	1.76 ± 0.167	4.31 ± 0.58
time-weighted mean*^a^*	—	0.150	0.312	0.754	1.76	4.30

% of nominal*^b^*	—	115	98	94	88	86

**flow-through test**
Range (min. - max.)	n.d.	0.06 – 0.16	0.23 – 0.30	0.52 – 0.73	1.29 – 1.50	3.01 – 4.09
mean ± SD	n.d.	0.13 ± 0.04	0.27 ± 0.03	0.62 ± 0.07	1.38 ± 0.10	3.45 ± 0.52

% of nominal*^c^*	—	100	84	78	69	69

*^a^* calculated according to OECD guideline 211, annex 6 (OECD, 2012)

*^b^* referring to measured time-weighted mean concentration

*^c^* referring to measured mean concentration
